# RECONSTRUCTING CULTURAL IDENTITY: THE ORAL NARRATIVES OF CENTENARIAN DESCENDANTS OF INDIGENOUS ANCESTRY

**DOI:** 10.1093/geroni/igae098.0384

**Published:** 2024-12-31

**Authors:** Alex Bishop, Tonya Finchum, Melinda Heinz

**Affiliations:** Oklahoma State University, Stillwater, Oklahoma, United States; Oklahoma State University, Stillwater, Oklahoma, United States; University of Northern Iowa, Cedar Falls, Iowa, United States

## Abstract

This study examined storytelling narratives of indigenous centenarians from the Oklahoma 100 Year Life Project. Qualitative content analysis was conducted using oral history narratives of N = 16 centenarian descendants (n = 5 men; n = 11 women) of American Indian ancestry (n = 8 White/Caucasian; n = 4 African-American; n = 4 Indigenous). Of interest was identifying themes reflecting cultural influences. Analyses resulted in four thematic classifications. The first theme included “transmission of historical trauma,” and involved resettlement, slavery, and race relations. One centenarian man reflected, “That’s the reason they’re called sooners... that use to belong to the Cherokees, and they took all the land...” A second theme involved “mutual interdependence.” One centenarian woman remarked, “I never enjoyed anything if I had it and didn’t share with somebody else. That’s the way I’ve been all my life. I just love to share things with people” A third theme concerned “leisure and sporting.” One centenarian man recounted training with the varsity track team, “... everybody was blowing just like a steam engine except me...I had been running all summer long out there in the buffalo grass pastures.” The fourth theme entailed “lifelong learning.” One centenarian woman recalled, “I organized a club... we studied Indian customs... our families and traditions... we called ourselves ‘Tsa-la-gi,’ which means Indian ladies.” Results will further highlight differential influences of American Indian cultural heritage on socio-emotional well-being in human longevity.

